# Association between coffee intake and skeletal muscle mass among U.S. adults: a population-based study

**DOI:** 10.3389/fnut.2024.1390309

**Published:** 2024-08-07

**Authors:** Huangyi Yin, Wei Zhu, Liuqing Guo, Weishan Li, Min Liang

**Affiliations:** ^1^The First Affiliated Hospital of Guangxi Medical University, Nanning, China; ^2^Geriatric Endocrinology, The First Affiliated Hospital of Guangxi Medical University, Nanning, China

**Keywords:** caffeine intake, caffeinated coffee, decaffeinated coffee, skeletal muscle mass, obesity

## Abstract

**Background:**

A limited number of studies have reported that the possible effects of coffee intake on skeletal muscle mass, but the results have been inconsistently conclusive and there are no large sample studies concerning the U.S. population. Therefore, the purpose of our study was to explore the connection between coffee consumption and skeletal muscle mass in U.S. adults.

**Methods:**

The population for this cross-sectional study was drawn from the National Health and Nutrition Examination Survey (NHANES) from 2011 to 2018. Appendicular lean mass was accurately obtained from DXA, and skeletal muscle mass was assessed using appendicular skeletal muscle mass adjusted for body mass index (ASMBMI). Coffee and caffeine consumptions were obtained on a 24-h dietary recall questionnaire. Furthermore, the associations between coffee and caffeine intake and skeletal muscle mass were evaluated using three multiple linear regression models and smoothed curve fitting. Subgroup analyses based on age, gender, ethnicity and body mass index (BMI) were performed to assess the robustness of these relationships.

**Results:**

This cross-sectional survey included a total of 8,333 participants. After adjusting for all covariates, higher intake of coffee, caffeinated coffee, and caffeine was associated with elevated ASMBMI (coffee: β = 0.01, 95% CI: 0.01, 0.02, *P*-value < 0.001; caffeinated coffee: β = 0.01, 95% CI: 0.01, 0.02, *P*-value < 0.001; caffeine: β = 0.02, 95% CI: 0.01, 0.04, *P*-value < 0.001). Meanwhile, smoothed curve fitting showed that coffee, caffeinated coffee, and caffeine intake were linearly and positively associated with ASMBMI. After further stratification by sex, age, and ethnicity, the positive relationships between coffee (especially caffeinated coffee) and caffeine intake and ASMBMI were not modified (*P* for interaction > 0.05). However, these relationships disappeared when the BMI over 30 kg/m^2^.

**Conclusions:**

In general, consumption of coffee and caffeine is positively associated with skeletal muscle mass. Therefore, an appropriate increase in coffee and caffeine intake may be advocated in populations at high risk for low skeletal muscle mass.

## Introduction

Sarcopenia is a chronic and progressive disease characterized by impairments in skeletal muscle mass and function. It is prevalent among the elderly and is an important contributor to the increase in falls, fractures, and all-cause mortality in the elderly, posing a serious threat to public health and safety (1–3). Epidemiological investigations have shown that muscle mass declines at a rate of 1%−2% per year in people over 50 years old, and the prevalence of sarcopenia reaches 40% in those over 80 years old (4). The development of sarcopenia has been reported to involve pathological processes such as chronic inflammation, decreased number and function of myosatellite cells, and oxidative stress (5–7).

According to the report of National Coffee Association, Americans consume an average of 517 million cups of coffee per day, and more than 60% of U.S. adults drink coffee daily (8). Coffee, one of the most popular beverages, is rich in caffeine and polyphenols, which have been shown to have antioxidant and anti-inflammatory activity and are involved in the energy metabolism processes (9–11). Researchers have conducted numerous population-based studies on the biological activities of caffeine and polyphenols. The results indicate that coffee intake may be beneficial to the prevention and treatment of cardiovascular disease, neurodegenerative diseases, diabetes mellitus (DM), cancers, and the reduction of all-cause mortality (12–15). However, coffee consumption may also adversely affect pregnancy outcomes and sleep quality (16).

The effects of coffee on skeletal muscle are still unclear. Considering the biological properties of coffee components, it is thought that coffee may improve skeletal muscle mass through mechanisms such as promoting autophagy, anti-inflammatory and antioxidant effects, and increasing the proliferation and differentiation potential of myosatellite cells (5–7). Researchers have investigated the link between coffee intake and sarcopenia, and varying results have appeared. One study in elderly Koreans reported a negative association between coffee intake and the prevalence of sarcopenic obesity, but not with sarcopenia or obesity alone (17). Other studies have suggested that increased coffee intake would be beneficial in improving muscle mass, reducing the incidence of sarcopenia, and this effect may vary by gender (18–20). Although several relevant studies have been conducted, their applicability is limited due to their primary focus on Japanese and Korean populations with small sample sizes. The relationship between coffee intake, caffeine consumption, and the prevalence of low muscle mass, particularly among Americans, remains unclear.

The purpose of this study was to explore the associations of caffeine and coffee intake (both caffeinated and decaffeinated) with skeletal muscle mass using the National Health and Nutrition Examination Survey (NHANES). This study is expected to further provide a promising theoretical foundation for guiding coffee and caffeine intake in populations at increased risk for low muscle mass.

## Methods

### Data sources and study population

NHANES is conducted by the National Center for Health Statistics (NCHS), which aims at collecting information on the health status and nutrition of Americans using a complex multistage, stratified probability sampling methodology. The study protocol was reviewed by the NCHS Research Ethics Board. Specific NHANES sample and data information is publicly available at https://www.cdc.gov/nchs/nhanes/.

**Figure 1:**
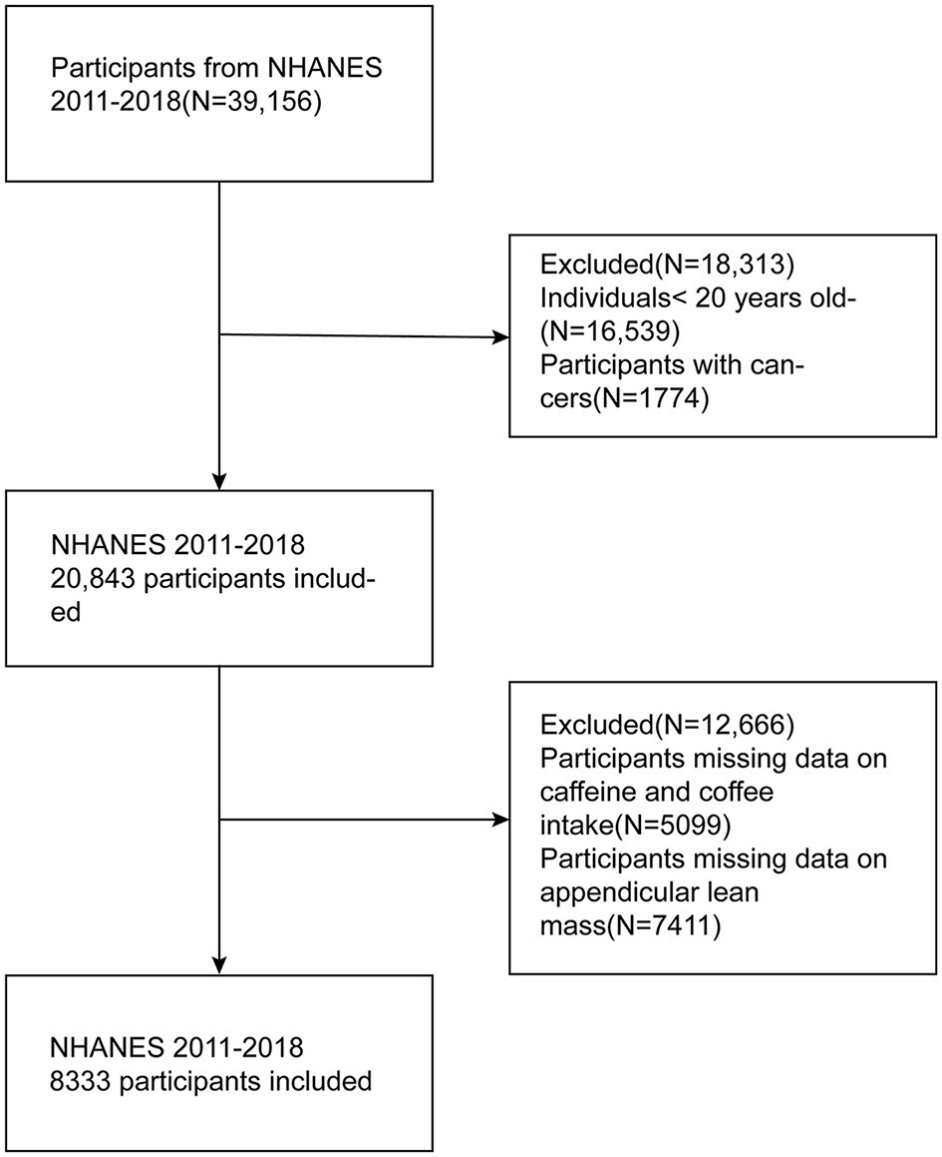
The flowchart of participants selection.

A total of 39,156 participants were interviewed during 2011–2018. We excluded participants based on the following criteria: (1) participants younger than 20 years old; (2) participants with missing data on appendicular lean mass (ALM); (3) adults who did not complete two 24-h dietary recall interviews or lacked data on coffee and caffeine intake; (4) participants who were ever told by their doctor that they had a cancer. It is worth mentioning that NHANES did not require completion of DXA for those over 60 years old. Ultimately, 8,333 participants were gathered in this cross-sectional study. The process is completely shown in Figure 1.

### Assessment of skeletal muscle mass

The Foundation for the National Institutes of Health (FNIH) recommends appendicular skeletal muscle mass adjusted by body mass index (ASMBMI) as important indicator for assessing sarcopenia; ASMBMI will be applied to evaluate skeletal muscle mass in this study. ASMBMI was calculated according to the following formula: ASMBMI (m^2^) = ALM (kg) of all four limbs/BMI (kg/m^2^). ALM was obtained by whole body DXA scanning.

### Evaluation of coffee and caffeine intake

The 24-h Dietary Recall Interview recorded detailed dietary intake information, including foods, beverages, and dietary supplements, for NHANES participants in the 24 h before the interview. All subjects were invited to participate in two dietary recall interviews. The first interviews were conducted at the mobile examination center (MEC) and the second interviews were conducted by telephone after 3–10 days. The energy and nutrient content of all foods and beverages was then calculated using the USDA Food and Nutritional Database for Dietary Studies (FNDDS). The total caffeine contained in foods, beverages and dietary supplements recorded at the two nonconsecutive 24-h dietary recall interviews was used to calculate the mean of the caffeine intake. Subjects who did not participate or who participated in only one interview were excluded. Caffeine intake was categorized into quartiles. Since most caffeine is derived from coffee, we further assessed coffee consumption (including both caffeinated and decaffeinated coffee) using the 24-h dietary recall questionnaire. Due to many participants not drinking coffee, we categorized these participants into Group 1, while the remaining participants were divided into Groups 2–4 based on tertiles of coffee consumption. As is shown in the Table 1.

**Table 1:** Grouping information according to the consumption of coffee and caffeine.

**Variables**	**Group 1**	**Group 2**	**Group 3**	**Group 4**
Coffee intake (g/day)	0	≤ 240.0	240.0–450.0	>450.0
Caffeinated hangindent8pt coffee intake hangindent8pt (g/day)	0	≤ 236.8	236.8–448.8	>448.8
Decaffeinated hangindent8pt coffee intake hangindent8pt (g/day)	0	≤ 154.8	154.8–292.5	>292.5
Caffeine intake (mg/day)	≤ 25.5	25.5–88.0	88.0–183.0	>183.0

### Covariates

According to previous research, we collected variables that may influence the relationship between coffee and caffeine intake and skeletal muscle mass. Data on demographic characteristics include sex (male, female), age, race (Mexican American, non-Hispanic white, non-Hispanic black, other), educational level (below high school, high school graduate, college or above), poverty income ratio (PIR) (< 1.3, 1.3–3.5, >3.5), and marital status (married or living with partners, single). Mean energy and protein intake from two 24-h dietary recall interviews were applied in this study. Body mass index (BMI) was calculated as weight (kg)/height (m)^2^ and then categorized into three groups: < 25, 25–30, ≥30 kg/m^2^. In addition, we obtained experimental data on albumin, vitamin D3, glutamic oxalic aminotransferase (AST), alanine aminotransferase (ALT), and creatinine. Finally, questionnaire data on smoking, alcohol consumption, physical activity (PA), DM, hypertension, and hyperlipidemia were included in this study.

Participants who smoked more than 100 cigarettes in their lifetime were defined as smoking status. The definition of drinking habits required a gender-based approach; except for non-drinkers, women were defined as moderate or heavy drinkers based on their consumption of 1 cup/day or ≥ 2 cups/day, respectively, while men were classified based on 1–2 cups/day or ≥3 cups/day. PA was assessed through energy expenditure, with information derived from the Physical Activity (PAQ_G) questionnaire, calculated as follows: time of exercise per day (min/day) ^*^ frequency of exercise per week (days/week) ^*^ recommended metabolic equivalent (MET) (21). Hyperlipidemia and DM were determined by asking participants whether a doctor had ever diagnosed them with hyperlipidemia or DM. Hypertension was defined as meeting ≥1 of the following criteria: (1) systolic blood pressure ≥130 mmHg and/or diastolic blood pressure ≥80 mmHg, with blood pressure being the average of three measurements; (2) self-reported hypertension.

### Statistical analysis

Sample weights provided by NHANES were considered for statistical analyses in this study to ensure that the results were representative of the U.S. noninstitutionalized population. Continuous variables were expressed as mean (standard deviation), and differences between groups were detected using Kruskal–Wallis *H*-test. Meanwhile, categorical variables were expressed as frequencies (percentages), and differences between groups needed to be assessed using chi-square tests. To further evaluate the association of coffee and caffeine intake with skeletal muscle mass, three weighted multiple linear regression models were created. Model 1 was not adjusted for any variables. Model 2 was adjusted for gender, age, and race. Model 3 was adjusted for gender, age, race, BMI, waist circumference (WC), PIR, education level, marital status, smoking status, alcohol consumption, PA, hypertension, DM, hyperlipidemia, total energy and protein intake, vitamin D3, albumin, AST, ALT, and creatinine. In addition, the potential linear relationship between coffee and caffeine intake, and ASMBMI was further investigated using smoothed curve fitting. Finally, subgroup analyses stratified by age, gender, ethnicity, and BMI were performed to fully explore the stability of these associations. In this study, we used EmpowerStats and R software for statistical analysis; *P*-value < 0.05 was considered statistically significant.

## Results

### Baseline characteristics of participants

As shown in Table 2, a total of 8,333 subjects were enrolled in this study from 2011 to 2018, including 49.21% males and 50.79% females, with the mean age of 39.57 (11.78) years. The mean intakes of caffeine and coffee were 162.10 (184.79) mg/day and 284.15 (427.21) g/day, of which the mean consumptions of caffeinated and decaffeinated coffee were 267.26 (419.86) g/day and 16.88 (93.63) g/day, respectively. Participants in quartile 4 of ASMBMI had higher intake of coffee, caffeinated coffee, and caffeine compared to those with quartile 1. In addition, compared to quartile 1 of the ASMBMI, quartile 4 participants tend to be male, younger, better educated, single, non-obese, with smaller WC, more PA, moderate alcohol consumption, less likely to develop hyperlipidemia and hypertension. They also exhibited higher levels of albumin, AST, ALT, creatinine, vitamin D3, and intakes of protein and energy. Although the incidence of DM was higher in quartile 4 than in quartile 1, it was significantly elevated in quartile 2 and quartile 3 relative to quartile 1, potentially due to more missing DM data in quartile 4.

**Table 2:** Baseline characteristics of participants.

**Variables**	**ASMBMI (m** ^**2**^ **)**				***P-*value**
	** ≤ 0.63 (*n* = 2,083)**	**0.63–0.78 (*n* = 2,083)**	**0.78–0.95 (*n* = 2,083)**	**>0.95 (*n* = 2,084)**	
Age (years)	41.91 (11.57)	38.94 (11.72)	40.12 (11.72)	37.70 (11.71)	< 0.001
**Gender (%)**					< 0.001
Male	17 (0.83)	261 (12.51)	1,570 (75.36)	2,058 (98.77)	
Female	2,066 (99.17)	1,822 (87.49)	513 (24.64)	26 (1.23)	
**Race (%)**					< 0.001
American Mexican	347 (16.64)	163 (7.84)	224 (10.76)	116 (5.58)	
Non-Hispanic White	1,157 (55.56)	1,307 (62.76)	1,326 (63.65)	1,360 (65.28)	
Non-Hispanic Black	164 (7.90)	283 (13.58)	176 (8.46)	332 (15.92)	
Others	415 (19.91)	330 (15.83)	357 (17.13)	276 (13.23)	
**Education level (%)**					< 0.001
Below high school	344 (16.53)	208 (9.97)	250 (11.98)	210 (10.08)	
High school graduate	511 (24.52)	400 (19.21)	441 (21.17)	445 (21.33)	
College or above	1,228 (58.95)	1,475 (70.82)	1,392 (66.85)	1,429 (68.59)	
**Marital status (%)**					0.005
Married/living with partner	1,362 (65.40)	1,263 (60.64)	1,350 (64.81)	1,299 (62.34)	
Single	721 (34.60)	820 (39.36)	733 (35.19)	785 (37.66)	
**PIR (%)**					< 0.001
< 1.3	546 (26.20)	446 (21.44)	418 (20.09)	363 (17.40)	
1.3–3.5	711 (34.14)	688 (33.02)	659 (31.63)	653 (31.34)	
>3.5	687 (32.99)	828 (39.74)	869 (41.73)	960 (46.08)	
Unknown	139 (6.67)	121 (5.81)	136 (6.55)	108 (5.18)	
**BMI (%)**					< 0.001
< 25 kg/m^2^	309 (14.85)	785 (37.67)	613 (29.43)	829 (39.76)	
25–30 kg/m^2^	573 (27.52)	600 (28.79)	623 (29.93)	860 (41.28)	
≥30 kg/m^2^	1,201 (57.64)	699 (33.54)	846 (40.63)	395 (18.95)	
WC (cm)	102.70 (16.64)	95.20 (16.92)	99.90 (16.60)	93.72 (12.65)	< 0.001
PA (MET^*^min/week)	2,040.36 (6,471.35)	2,729.68 (8,950.69)	3,190.08 (4,865.71)	4,455.89 (23,202.79)	< 0.001
**Drink status (%)**					< 0.001
Never	503 (24.07)	325 (15.63)	221 (10.64)	172 (8.28)	
Moderate	307 (14.71)	406 (19.49)	554 (26.60)	800 (38.40)	
Heavy	672 (32.16)	777 (37.35)	684 (32.88)	639 (30.67)	
Unknown	607 (29.07)	573 (27.53)	622 (29.88)	471 (22.64)	
**Smoking (%)**					< 0.001
No	1,387 (66.58)	1,317 (63.22)	1,153 (55.35)	1,207 (57.90)	
Yes	696 (33.42)	766 (36.76)	929 (44.60)	876 (42.07)	
Unknown	0 (0.00)	0 (0.02)	1 (0.05)	1 (0.03)	
**DM (%)**					< 0.001
No	1,918 (91.87)	1,969 (94.61)	1,923 (92.42)	1,817 (87.22)	
Yes	166 (7.94)	103 (4.94)	111 (5.33)	191 (9.15)	
Unknown	4 (0.19)	9 (0.45)	47 (2.25)	76 (3.63)	
**Hypertension (%)**					< 0.001
No	1,335 (64.10)	1,472 (70.68)	1,347 (64.67)	1,447 (69.41)	
Yes	717 (34.42)	590 (28.30)	704 (33.78)	616 (29.58)	
Unknown	31 (1.48)	21 (1.02)	32 (1.55)	21 (1.02)	
**Hyperlipidemia (%)**					< 0.001
No	1,490 (71.53)	1,633 (78.39)	1,469 (70.52)	1,594 (76.51)	
Yes	580 (27.84)	443 (21.27)	611 (29.34)	487 (23.37)	
Unknown	13 (0.63)	7 (0.35)	3 (0.14)	3 (0.13)	
Albumin (g/L)	41.36 (3.11)	42.41 (3.16)	43.53 (3.28)	43.77 (3.24)	< 0.001
ALT (U/L)	23.34 (18.50)	19.98 (12.36)	24.17 (15.15)	27.17 (18.31)	< 0.001
AST (U/L)	23.86 (20.78)	22.52 (15.19)	24.77 (11.62)	26.13 (11.51)	< 0.001
Creatinine (μmol/L)	62.72 (15.28)	68.72 (33.32)	81.90 (39.55)	85.41 (24.76)	< 0.001
Vitamin D3 (nmol/L)	60.15 (26.08)	65.46 (28.52)	64.04 (25.93)	64.80 (24.44)	< 0.001
Energy intake (kcal/day)	1,783.22 (611.77)	1,908.25 (679.78)	2,307.85 (825.47)	2,570.88 (892.15)	< 0.001
Protein intake (g/day)	69.16 (26.24)	73.63 (28.02)	90.28 (34.25)	101.99 (41.88)	< 0.001
Coffee intake (g/day)	235.37 (355.03)	278.28 (387.76)	312.62 (401.11)	302.64 (527.15)	< 0.001
Caffeinated coffee intake (g/day)	217.88 (351.90)	264.88 (385.10)	291.83 (393.62)	286.78 (514.05)	< 0.001
Decaffeinated coffee intake (g/day)	17.48 (81.89)	13.41 (66.42)	20.79 (106.79)	15.86 (109.54)	0.072
Caffeine intake (mg/day)	137.91 (155.35)	153.84 (163.02)	176.74 (181.46)	175.83 (223.36)	< 0.001

Continuous variables were expressed as mean (standard deviation), and categorical variables were expressed as frequencies (percentages).

PIR, poverty income ratio; BMI, body mass index; WC, waist circumference; PA, physical activity; MET, metabolic equivalent of task; DM, diabetes mellitus; AST, glutamic oxalic aminotransferase; ALT, alanine aminotransferase.

### The associations of coffee and caffeine intake with ASMBMI

The associations between coffee, caffeine intake and ASMBMI were explored using three multiple linear regression analysis models, as shown in Table 3. Coffee, caffeinated coffee, and caffeine intake were positively related to ASMBMI in all models. After adjusting for all covariates in Model 3, group 4 of coffee consumption was associated with a 13% increase in ASMBMI compared to group 1 (β = 0.13, 95% CI: 0.06, 0.19, *P*-value < 0.001). Similarly, caffeinated coffee intake in group 4 showed an association with an increase in ASMBMI compared to group 1 (β = 0.12, 95% CI: 0.06, 0.18, *P*-value < 0.001). In addition, caffeine intake in group 4 was associated with an 11% increase in ASMBMI compared to group 1 (β = 0.11, 95% CI: 0.04, 0.17, *P*-value = 0.003). Multifactorial regression analyses were not conducted for intake of decaffeinated coffee, as it did not show statistical significance across ASMBMI quartiles.

**Table 3:** Association of coffee and caffeine intake with ASMBMI.

**Variables**	**Model 1 [β (95% CI) *P-*value]**	**Model 2 [β (95% CI) *P-*value]**	**Model 3 [β (95% CI) *P-*value]**
Coffee intake (per 1,000 g)	0.02 (0.01, 0.03) < 0.001	0.02 (0.01, 0.02) < 0.001	0.01 (0.01, 0.02) < 0.001
**Coffee intake (groups)**		
Group 1	Reference	Reference	Reference
Group 2	−0.40 (−0.53, −0.28) < 0.001	0.01 (−0.06, 0.08) 0.852	0.01 (−0.06, 0.08) 0.763
Group 3	−0.10 (−0.22, 0.02) 0.115	0.23 (0.16, 0.30) < 0.001	0.07 (0.00, 0.13) 0.039
Group 4	0.11 (0.00, 0.22) 0.049	0.24 (0.17, 0.30) < 0.001	0.13 (0.06, 0.19) < 0.001
*P* for trend	0.073	< 0.001	< 0.001
Caffeinated coffee intake (per 1,000 g)	0.02 (0.01, 0.03) < 0.001	0.01 (0.01, 0.02) < 0.001	0.01 (0.01, 0.02) < 0.001
**Caffeinated coffee intake (groups)**		
Group 1	Reference	Reference	Reference
Group 2	−0.42 (−0.55, −0.29) < 0.001	0.03 (−0.04, 0.11) 0.391	0.05 (−0.02, 0.11) 0.170
Group 3	−0.01 (−0.13, 0.11) 0.929	0.18 (0.11, 0.25) < 0.001	0.04 (−0.03, 0.10) 0.256
Group 4	0.14 (0.04, 0.25) 0.009	0.22 (0.15, 0.28) < 0.001	0.12 (0.06, 0.18) < 0.001
*P* for trend	0.009	< 0.001	< 0.001
Caffeine intake (per 1,000 mg)	0.07 (0.05, 0.10) < 0.001	0.02 (0.01, 0.04) < 0.001	0.02 (0.01, 0.04) < 0.001
**Caffeine intake (groups)**		
Group 1	Reference	Reference	Reference
Group 2	−0.28 (−0.41, −0.14) < 0.001	−0.04 (−0.11, 0.04) 0.359	0.01 (−0.06, 0.08) 0.804
Group 3	−0.23 (−0.36, −0.10) < 0.001	−0.03 (−0.10, 0.04) 0.428	−0.04 (−0.10, 0.03) 0.275
Group 4	0.16 (0.04, 0.28) 0.009	0.13 (0.06, 0.21) < 0.001	0.11 (0.04, 0.17) 0.003
*P* for trend	< 0.001	< 0.001	0.006

Model 1 did not adjust for any covariates. Model 2 adjusted for gender, age, and race. Model 3 adjusted for all covariates.

**Figure 2:**
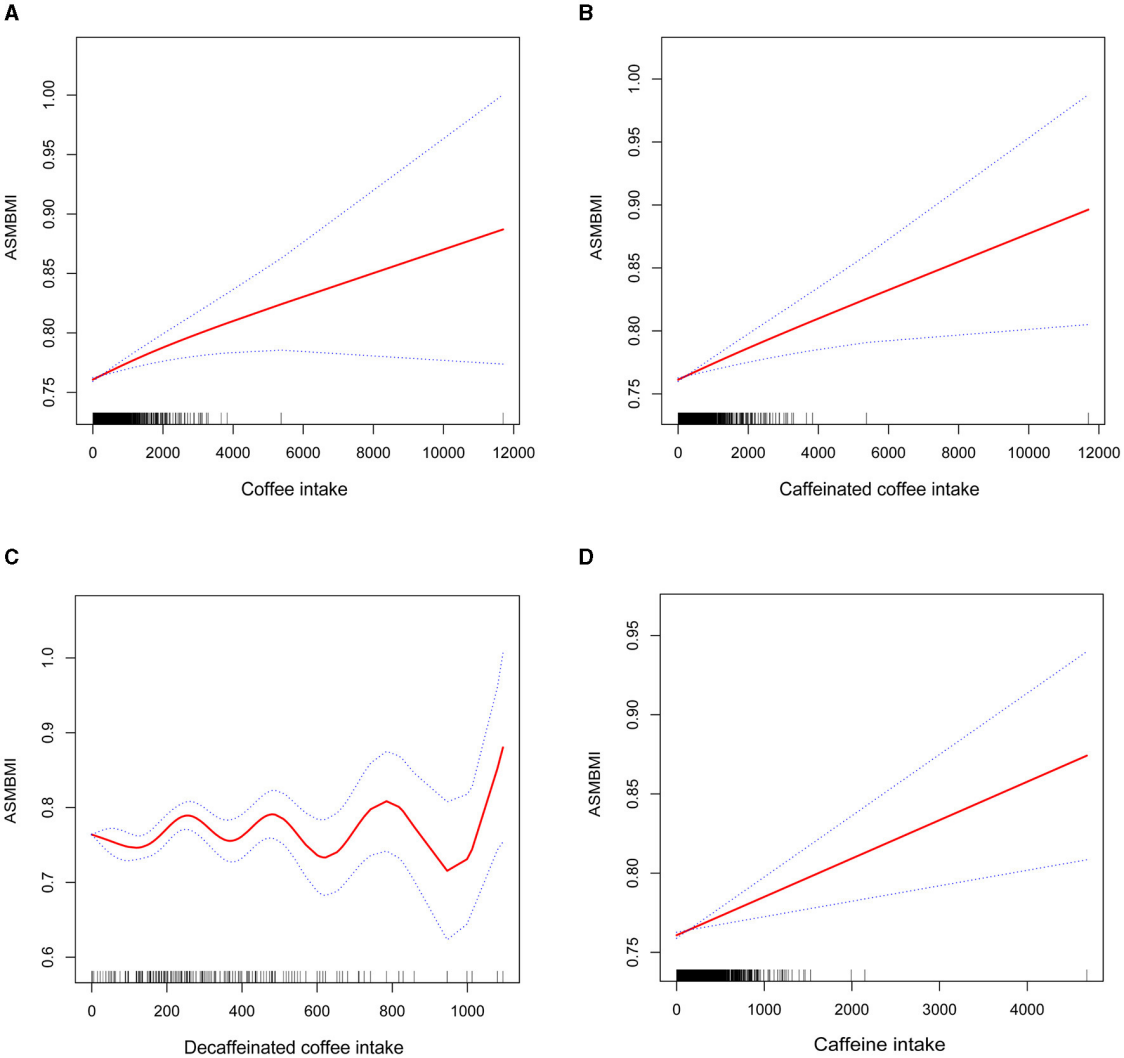
Smoothed curve fitting of coffee intake (including caffeinated coffee, decaffeinated coffee), caffeine intake and ASMBMI. **(A)** coffee intake, **(B)** caffeinated coffee intake, **(C)** decaffeinated coffee intake, and **(D)** caffeine intake.

In Figure 2, the smoothed curve fitting generalized model showed that intake of either caffeine, coffee or caffeinated coffee was positively associated with ASMBMI in an approximately linear fashion, whereas the relationship between decaffeinated coffee and ASMBMI showed a wavy pattern.

### Subgroup analysis

Subgroup analysis revealed no significant interaction between caffeine and coffee (including caffeinated coffee) intake with gender, age, or race. However, BMI affected the positive association of caffeine and coffee (including caffeinated coffee) intake with ASMBMI. Caffeine and coffee intake were not associated with ASMBMI when BMI was ≥30 kg/m^2^ (Table 4).

**Table 4:** Subgroup analyses for the relationship of coffee and caffeine intake with ASMBMI.

**Subgroups**	**Coffee^a^ [β (95% CI) *P-*value]**	***P* ^b^**	**Caffeinated coffee^a^ [β (95% CI) *P*-value]**	***P* ^b^**	**Caffeine^a^ [β (95% CI) *P*-value]**	***P* ^b^**
**Age (years)**		0.526		0.659		0.519
20–40	0.01 (−0.00, 0.02) 0.149		0.01 (−0.00, 0.02) 0.150		0.01 (−0.01, 0.04) 0.213	
40–59	0.01 (0.01, 0.02) < 0.001		0.01 (0.00, 0.02) 0.002		0.02 (0.01, 0.04) 0.003	
**Gender**		0.147		0.079		0.110
Male	0.01 (0.00, 0.02) 0.010		0.01 (0.00, 0.01) 0.041		0.02 (−0.00, 0.03) 0.059	
Female	0.02 (0.01, 0.03) < 0.001		0.02 (0.01, 0.03) < 0.001		0.04 (0.02, 0.06) < 0.001	
**Race**		0.765		0.633		0.895
American Mexican	0.01 (−0.02, 0.04) 0.699		0.00 (−0.03, 0.03) 0.931		0.01 (−0.05, 0.08) 0.713	
Non-Hispanic White	0.01 (0.01, 0.02) < 0.001		0.01 (0.00, 0.02) 0.002		0.02 (0.01, 0.04) 0.002	
Non-Hispanic Black	0.00 (−0.03, 0.03) 0.931		0.00 (−0.03, 0.03) 0.980		0.01 (−0.06, 0.07) 0.880	
Others	0.02 (0.01, 0.03) 0.004		0.02 (0.01, 0.03) 0.003		0.03 (0.00, 0.06) 0.024	
**BMI (kg/m** ^2^ **)**		0.001		< 0.001		0.003
< 25	0.03 (0.02, 0.04) < 0.001		0.02 (0.01, 0.03) < 0.001		0.05 (0.03, 0.07) < 0.001	
25–30	0.01 (0.01, 0.02) 0.001		0.01 (0.01, 0.02) 0.001		0.03 (0.01, 0.05) 0.002	
≥30	0.00 (−0.01, 0.01) 0.906		−0.00 (−0.01, 0.01) 0.741		−0.00 (−0.02, 0.02) 0.815	

^a^The unit of measure is divided by 1,000 from the original.

^b^*P* for interaction.

All covariates were adjusted. BMI, body mass index.

## Discussion

This study explored the association between caffeine, coffee intake, and skeletal muscle mass. After adjusting for all covariates, the positive associations of caffeine and coffee intake with ASMBMI persisted. These relationships were not affected by gender, age, or race but may be influenced by BMI.

Considering that coffee has already become the second most popular beverage in the world, its effects on human health have received attention from the scientific community (22). In recent years, researchers have investigated the relationship between coffee intake and muscle mass, sarcopenia, yielding diverse results. A study of Korean adults showed that men who drank one cup of coffee per day had a 31% reduction in sarcopenia compared to non-coffee drinkers, but this association was not observed in men who drank more than one cup of coffee daily or in women (23). The Korean National Health and Nutrition Examination Survey (KNHANES) showed that the prevalence of sarcopenia was as high as 9% among elderly men who drank < 1 cup of coffee per day, but it reduced to 3.6% when more than three cups of coffee per day were consumed (19). Skeletal muscle mass index (SMI), an important index for evaluating skeletal muscle mass, has been observed to positively correlate with coffee intake in middle-aged and elderly Japanese individuals. However, this study was not explored the correlation between coffee intake and muscle strength (20). Similar results were reported in another study, where coffee intake was negatively associated with sarcopenia diagnosed on the basis of ALM/height^2^ (18). These findings are consistent to our results that coffee or caffeine consumption correlates with increased skeletal muscle mass.

Notably, sarcopenia is strongly associated with obesity (24). The decrease in muscle mass accompanied by an increase in fat mass is defined as “sarcopenic obesity,” with the mechanisms potentially linked to the activation of the inflammatory response by cytokines produced by adipocytes, thereby increasing the catabolism of lean muscle mass (25, 26). A study based on KNHANES also elaborated on the relationship between coffee intake and sarcopenic obesity, finding that older adults who drank coffee < 1 cup per day were 5.861 times more likely to suffer from sarcopenic obesity than those who drank ≥3 cups of coffee per day. However, daily coffee intake was not associated with obesity alone or sarcopenia alone (17). Mechanistically, caffeine stimulates sympathetic nerves and increases energy metabolism in the body, while polyphenols may increase fat loss by modulating intestinal flora, thereby leading to weight loss (27–29). An observational study supported the relationship between coffee intake and obesity (30), but other studies suggest that habitual coffee consumption will increase the risk of obesity (23, 31). Our study indicated that higher consumption of coffee (including caffeinated coffee) or caffeine was associated with increased skeletal muscle mass at BMI < 30 kg/m^2^, but these relationships disappeared in obesity. This may be explained by the fact that the rate of lean muscle catabolism in obese patients has outpaced the beneficial effects of coffee intake on skeletal muscle. Therefore, we should carefully guide coffee intake in obese patients.

Several mechanisms may explain the positive correlation between coffee or caffeine intake and skeletal muscle mass: (1) Autophagy, an important pathway for maintaining mitochondrial degradation and recirculation, occurs at the highest rate in skeletal muscle and is influenced by exercise and starvation. It is essential for maintaining muscle mass and integrity (5). During aging, a decrease in autophagy will disrupt muscle homeostasis, leading to mitochondrial dysfunction, oxidative stress, and ultimately muscle weakness and atrophy (32, 33). In female mice, coffee was demonstrated to increase skeletal muscle autophagy in a dose-dependent manner (34). In fact, the main active ingredients of coffee, including caffeine and polyphenols, have also been shown to induce autophagy and increase the degradation of damaged proteins and organelles (4, 35). (2) Meta-analysis indicated that decreased skeletal muscle strength and muscle mass implied elevated circulating inflammatory markers, suggesting that chronic inflammatory responses may be involved in the reduction of skeletal muscle mass (36). Polyphenols and caffeine in coffee have been shown to possess anti-inflammatory and oxygen free radical scavenging properties, making them potential candidates for the improvement of skeletal muscle mass (37, 38). Guo et al. found that decreased muscle mass and strength in aged mice were effectively improved after 4 weeks of coffee intervention, accompanied by decreased levels of inflammatory mediators in the body (39). The function and number of myosatellite cells are important factors in maintaining muscle mass. A cellular assay indicated that coffee intervention increased the proliferation rate and DNA synthesis of human satellite cells through activation of the Akt signaling pathway (39). In addition, decreased expression of muscle growth inhibitor mRNA and elevated expression of insulin-like growth factor were also associated with coffee consumption, reflecting skeletal muscle hypertrophy and improved muscle function (40).

Sarcopenia is a serious health risk that may lead to falls, fractures, and even increased mortality. The results of this study have important implications for guiding daily coffee intake to prevent sarcopenia. However, there are several limitations in this cross-sectional study. Firstly, this study evaluated skeletal muscle mass using the ASMBMI without additional assessment the muscle strength in the subjects. However, according to previous studies, appropriate ASMBMI indicators can also contribute to diagnose sarcopenia (41, 42). Secondly, although we were not able to separately categorize sarcopenic obesity, we included covariates of anthropometric indicators used to assess obesity (such as BMI, WC) to minimize the errors caused by small numbers of obese individuals. Thirdly, the data on coffee and caffeine intake were derived from the 24-h dietary recall interview, which failed to detect levels of caffeine *in vivo* and in the diet, potentially introducing recall bias. Fourthly, the present study is a large cross-sectional study based on the NHANES database and thus cannot infer a causal relationship between coffee and caffeine intake and muscle mass.

However, our study has certain strengths. This is a large sample cross-sectional study that performed a weighted analysis to obtain findings representative of the U.S. citizens. In addition, we stratified the data according to gender, age, race and BMI, providing valuable insights for guiding individualized dietary regimens for patients with reduced skeletal muscle mass. Therefore, we should be cautious in interpreting the results and set up a longitudinal cohort study to further explore the causal relationship between coffee intake and skeletal muscle mass.

## Conclusions

In conclusion, higher coffee and caffeine consumption was related to increasing skeletal muscle mass. This study emphasizes the potentially significant value of coffee and the caffeinated food intake in reducing the risk of low muscle mass in adults.

## Data availability statement

The original contributions presented in the study are included in the article/supplementary material, further inquiries can be directed to the corresponding author.

## Ethics statement

The studies involving humans were approved by the National Center for Health Statistics Research Ethics Board. The studies were conducted in accordance with the local legislation and institutional requirements. The participants provided their written informed consent to participate in this study.

## Author contributions

HY: Data curation, Formal analysis, Methodology, Software, Writing – original draft. WZ: Data curation, Formal analysis, Methodology, Writing – original draft. LG: Methodology, Visualization, Writing – original draft. WL: Methodology, Visualization, Writing – original draft. ML: Supervision, Writing – review & editing.
